# The Prevalence and the Associated Sociodemographic-Occupational Factors of Professional Burnout Among Health Professionals During COVID-19 Pandemic in Malang, Indonesia: A Cross-Sectional Study

**DOI:** 10.3389/fpubh.2022.894946

**Published:** 2022-07-01

**Authors:** Besut Daryanto, Frilya Rachma Putri, Jemmy Kurniawan, Muhammad Ilmawan, Jonny Karunia Fajar

**Affiliations:** ^1^Department of Urology, Faculty of Medicine, Universitas Brawijaya, Malang, Indonesia; ^2^Department of Psychiatry, Faculty of Medicine, Universitas Brawijaya, Malang, Indonesia; ^3^Brawijaya Internal Medicine Research Center, Faculty of Medicine, Universitas Brawijaya, Malang, Indonesia

**Keywords:** professional burnout, COVID-19, health occupations, prevalence studies, Indonesia

## Abstract

**Background:**

Since 2020, Indonesian health professionals have been affected by burnout as the physiological impact due to the COVID-19 pandemic. Malang has contributed to a substantial number of new daily cases and death rates in East Java, an epicenter of COVID-19 in Indonesia. However, a study about how burnout affected Malang health professionals was never conducted.

**Objectives:**

This research aimed to assess the prevalence and factors associated with burnout among health professionals during the COVID-19 pandemic in Malang, Indonesia.

**Materials and Methods:**

A cross-sectional study was carried out with an online survey conducted in 5 major hospitals in Malang. We conducted a study about the prevalence rate of burnout in Malang and the association between sociodemographic factors, occupational hazards, and the Maslach Burnout Inventory-Human Services Survey (MBI-HSS). The associations were presented as odds ratio (*OR*) and 95% confidence interval (*CI*).

**Results:**

We analyzed 1,077 health professionals in Malang. Our result showed that the prevalence of burnout among health professionals in Malang is 22.0%. Respondents under the age of 30 tend to experience a higher level of burnout by 3.4-fold (*OR* = 3.43, *p*-value < 0.001), compared with those over the age of 40 years. Our data showed that respondents working longer than 100 h/week tend to experience burnout by 3.8-fold (*OR* = 3.83, *p*-value < 0.001).

**Conclusion:**

Approximately one-fifth of the health professionals in Malang suffered from burnout during the COVID-19 pandemic, and MBI-HSS subscales are strongly associated with age and work hours.

## Introduction

The World Health Organization (WHO) declared coronavirus disease 2019 (COVID-19) a pandemic on March 11, 2020. By April 9, 2020, COVID-19 had spread across all 34 provinces in Indonesia and subsequently reached 56,757 cases in a day on July 15, 2021, the largest of new COVID-19 cases in the world on that day. During this period, half of Indonesia's provinces had a more than 50% increase in COVID-19 cases, and East Java has the highest death rate of all provinces. In August 2021, the overall number of patients with COVID-19 in Malang, as the most populated area in East Java, remained high even after a local lockdown was held (1–3). This condition led to an overwhelming impact on Malang health professionals who are at the greatest risk of being infected. Health professionals had to continue their services in the hospital with constrained resources and precarious infrastructure. They must wear personal protective equipment (PPE), which leads to physical discomfort and breathing difficulty. In addition, they also need to be more cautious about the possibility of transmitting the virus to their family (4–7). These behavioral changes in daily life have been identified as factors that have a detrimental psychological influence on health professionals (8). A recent systematic review showed that one-third of Asian health providers suffered from psychological distress and other psychiatric disorders during the pandemic (9). These conditions may put health professionals in a burnout condition.

Burnout is a work-related psychological syndrome characterized by emotional exhaustion (EE), depersonalization (DP), and reduced sense of personal accomplishment (PA) (10). Burnout among health professionals has been linked to a greater risk of depression, anxiety, drug abuse, medical errors, and poor clinical decision-making leading to compromised personal wellbeing and patient safety (11–13). Amid the outbreaks of severe acute respiratory syndrome (SARS), H1N1, and Ebola, several studies showed that psychological sequelae were more likely to be found in frontline health professionals (13). However, when compared with the previous multinational endemics, the consequence of the COVID-19 pandemic was more serious.

Although burnout has increased among health professionals during the COVID-19 pandemic, a study about burnout in Malang has not been done yet. The primary objectives of this study were to evaluate the prevalence of burnout among health professionals and to identify the factors that contributed to burnout during the COVID-19 pandemic. We hypothesized that health professionals in Malang may have burnout during COVID-19 pandemic as in other countries, and sociodemographic factors and occupational hazards may contribute to those condition. A better understanding of the associated factors may improve how health professionals and health organizations face the horror of the COVID-19 pandemic.

## Materials and Methods

### Study Design

We conducted a cross-sectional study to determine the prevalence rate and factors associated with burnout among health professionals during the COVID-19 pandemic in Malang. The definition of healthcare professionals in our study was a person that applies scientific knowledge relating to medicine as follows: (1) medical doctors; (2) nurses; and (3) other health professionals (14). To assess the prevalence rate, the definition of burnout used in our study was based on the Maslach Burnout Inventory for Human Service and its three subscales (EE, DP, and PA) (15). The correlation among sociodemographic factors, work-related factors, and each of the Maslach Burnout Inventory-Human Services Survey (MBI-HSS) subscale categories was calculated as odds ratio (*OR*) and 95% confidence interval (*CI*). Strengthening The Reporting of Observational Studies in Epidemiology (STROBE) checklist was used to ensure our study quality (16).

### Data Collection

A survey questionnaire was used to collect data from health professionals from 5 COVID-19 referral hospitals in Malang, Indonesia. The health professionals from public hospitals were selected from Saiful Anwar Hospital and Kanjuruhan Hospital. Meanwhile health professionals participating from private hospitals in this study were selected from Persada Hospital, Panti Waluya Hospital, and Wava Husada Hospital. Those hospitals were chosen based on the similarity of bed occupancy rate of COVID-19 services. Furthermore, the survey was conducted from August 1 to 31, 2021 using the Google Form platform, which was distributed to the representatives of each hospital together with information about the study procedures, ethical issues, and data collection. The required sample size was calculated using Cochran's formula estimating a 56.67% burnout prevalence in Saiful Anwar Hospital (17). Power was set at 80% and significance at 0.05. A minimal sample size was calculated at *n* = 377 for burnout healthcare professionals. Sample size was obtained using non-probability convenient sampling technique and adequate sample sizes were obtained according to sample size calculation. Larger numbers have been included to increase power for sub-analyses. Afterward, the results of the data gathering process were processed by 2 independent authors (JK and MI) to ensure its validity and confidentiality.

### Eligibility Criteria

From the obtained responses, we only included samples that met the following inclusion criteria: (1) working as a health professional; (2) agreeing to participate; and (3) participating in COVID-19 services. However, exclusion criteria in our study were (1) healthcare professional who are currently not working in the designated hospital and (2) duplicate response.

### Instruments

A questionnaire survey consisting of 34 questions was used in this study, including 1 question about identity, 2 questions about survey agreement, 4 questions about sociodemographic characteristics, 5 questions about occupational characteristics, and 22 questions of MBI-HSS in Indonesian language (18). The questions about identity, agreement, sociodemographic characteristics, and occupational characteristics were the combination of the open and close question models. While on MBI-HSS questions, the 7-point Likert scale was used ranging from 0 for “never” to 7 for “every day.” MBI-HSS questions have 3 subscales consisting of 9 questions about EE, 5 questions about DP, and 8 questions about PA, and each subscale has its unique level categories sorted from “low” (EE ≤ 16; DP ≤ 6; and PA ≤ 31), “moderate” (EE = 17–26; DP = 7–12; PA = 32–38), and “high” (EE ≥ 27; DP ≥ 13; and PA ≥ 39). The definitions of burnout were still not having consensus yet, we agreed to defined a burnout condition in our study as people who experienced “exhaustion” with a high level of EE or “cynicism” with a high level of DP based on several previous studies (19–21). EE was a condition described as an individual with depleted emotional resources and no longer able to care for themselves at a psychological level, and cynicism was described as the development of negative and cynical attitudes and feelings toward people (15).

### Ethical Considerations

All participants have been given informed consent and agreement in the early section of the online questionnaire survey. Our study was conducted according to the Declaration of Helsinki, and ethical approval was obtained from the Ethics Committee of Saiful Anwar General Hospital (Ref 400/083/K.3/302/2021 on April 19, 2021). Voluntary participation and data confidentiality were emphasized.

### Statistical Analyses

Our study calculated the correlation between sociodemographic, occupational characteristics, and burnout depending on each subscale using statistical analysis. Sociodemographic factors, occupational hazards, and burnout categories were processed as nominal data and the MBI subscale was processed as ordinal data. The relationship between burnout category and the independent variables were calculated using binomial logistic regression, and ordinal logistic regression was used to calculate the relationship between MBI subscales and the associated factors. The results of the statistical calculation shown as *OR* and *OR*95% *CI*. The test used above is two-sided and the *p*-value is considered significant if it is <0.05. In addition, Cronbach's alpha (α) was calculated in our statistical analysis to see the reliability of the Indonesian version of the MBI-HSS questionnaire. All statistical tests in our study were conducted using SPSS version 23 (IBM Corp. Released 2015. IBM SPSS Statistics for Windows, Version 23.0. Armonk, NY: IBM Corp).

## Results

### Baseline Characteristics

Our study involved 1,077 health professionals who worked during the COVID-19 pandemic in Malang. All respondents agreed to participate, but 15 pieces of data from our respondents cannot be used because of duplicate responses. Therefore, sociodemographic characteristics of the respondents showed that our study involved more women (65.6%) than men (34.4%) with an average age of 34 years old. Most of the respondents were married (75.5%) and lived with their families (52.5%). While from occupational characteristics, the professions included in our study were the nurses (51.0%), doctors (39.6%), and others (9.4%) who worked in public hospitals (59.0%) and private hospitals (41.0%). Most of the respondents worked <70 h/week and they work on non-emergency (72.3%) and emergency service (55.2%), only if one person could work on more than 1 duty. Furthermore, the information about sociodemographic and occupational characteristics used in our study is shown in Table 1.

**Table 1:** Sociodemographic and occupational characteristics of the study sample (*N* = 1,077).

**Characteristics**	***N* (%)**
**Sex**
Male	371 (34.4)
Female	706 (65.6)
**Age (year)**	33.8 (8.2)
<30	398 (37.0)
30–40	477 (44.3)
>40	202 (18.8)
**Marital status**
Married	813 (75.5)
Not married	264 (24.5)
**Living**
Alone (home)	310 (28.8)
Alone (rent)	202 (18.8)
With family/parents	565 (52.5)
**Profession**
Doctor	427 (39.6)
Nurse	549 (51.0)
Others	101 (9.4)
**Hospital sector**
Public	635 (59.0)
Private	442 (41.0)
**Work hours (hour/week)**
<70	777 (72.1)
70–100	266 (24.7)
>100	34 (3.2)
**Workload**
**Emergency service**
Yes	595 (55.2)
No	482 (44.8)
**Non-emergency service**
Yes	779 (72.3)
No	298(27.7)
**Administrative**
Yes	61 (5.7)
No	1,016 (94.3)

*Mean (standard deviation)*.

### Burnout Prevalence Based on MBI-HSS Subscales

Our result showed that the prevalence of burnout among health professionals in 5 major hospitals in Malang is 22.0%. That result is accumulated from 9.8% of respondents with a high level of DP and 20.6% of respondents with a high level of EE. Therefore, a low level of PA is shown on 4.9% of our respondents. Moreover, our results also showed that the internal consistency of the MBI-HSS Indonesian version is more than 0.7 that is interpreted as adequate for subscales EE (α = 0.881), DP (α = 0.807), and PA (α = 0.783). Detailed information on the prevalence of burnout for each subscale is presented in Table 2.

**Table 2:** Distribution of the Maslach Burnout Inventory-Human Services Survey (MBI-HSS) subscale scores and the prevalence of burnout.

**Indicators**	***N* (%)**	**Mean**	**SD**	**Cronbach's α**
**Burnout (high EE or DP)**	237 (22.0)			
**EE**				0.881
Low (0–16)	577 (53.6)	9.1	4.629	
Moderate (17–26)	278 (25.8)	21	2.778	
High (≥27)	222 (20.6)	34.67	5.999	
**DP**				0.807
Low (0–6)	808 (75.0)	4.39	5.258	
Moderate (7–12)	163 (15.1)	8.9	1.605	
High (≥13)	106 (9.8)	16.79	3.685	
**PA**				0.783
Low (0–31)	53 (4.9)	50.42	9.887	
Moderate (32–38)	83 (7.7)	35.4	1.944	
High (≥39)	941 (0.9)	53.12	7.006	

*EE, Emotional exhaustion; DP, depersonalization; PA, Personal accomplishment*.

### The Factors Associated With Burnout

Several factors from sociodemographic and occupational hazards associated with burnout on health professionals during the COVID-19 pandemic were presented in our result. Our result showed that the associated sociodemographic factors of burnout were male gender, younger age, and not in marital commitment (*OR* = 1.47, *p*-value = 0.015; *OR* = 3.43, *p*-value < 0.001; and *OR* = 1.50, *p*-value = 0.042). The Associated occupational hazards of burnout were medical professionals, working in a private hospital, and long work hours (*OR* = 2.78, *p*-value < 0.001; *OR* = 2.92, *p*-value < 0.001; and *OR* = 3.83, *p*-value < 0.001). The detailed results of burnout associated factors are presented in Table 3.

**Table 3:** The logistic regression odds ratio (*OR*) and 95% confidence interval (*CI*) of burnout and each of the MBI subscale scores compared by sociodemographic and occupational hazards.

	**Burnout**				**EE**				**DP**				**PA**			
	**OR**	**Lower OR**	**Upper OR**	***p*-value**	**OR**	**Lower OR**	**Upper OR**	***p*-value**	**OR**	**Lower OR**	**Upper OR**	***p*-value**	**OR**	**Lower OR**	**Upper OR**	***p*-value**
		**95% CI**	**95% CI**			**95% CI**	**95% CI**			**95% CI**	**95% CI**			**95% CI**	**95% CI**	
**Sociodemographic factors**
Male vs. female	1.47	1.08	2.00	0.015*	1.33	1.04	1.7	0.022*	1.60	1.20	2.14	0.001*	1.15	0.78	1.68	0.481
**Age (year)**
<30 vs. >40	3.43	1.90	6.21	<0.001*	2.17	1.48	3.18	<0.001*	3.14	1.82	5.42	<0.001*	1.15	0.62	2.15	0.654
30–40 vs. >40	3.51	2.01	6.14	<0.001*	2.11	1.49	2.99	<0.001*	3.74	2.25	6.23	<0.001*	1.56	0.89	2.71	0.118
**Living companion:**
Alone (rent) vs. alone (home)	1.21	0.76	1.94	0.418	1.07	0.82	1.41	0.615	0.84	0.60	1.18	0.316	0.86	0.56	1.34	0.510
With family vs. alone (home)	1.04	0.72	1.49	0.848	1.23	0.84	1.79	0.286	1.05	0.68	1.64	0.813	1.34	0.77	2.36	0.302
Not married vs. married	1.50	1.02	2.22	0.042*	1.39	1.01	1.92	0.045	1.41	0.97	2.06	0.073	1.31	0.79	2.15	0.294
**Occupational hazards**
**Profession:**
Others vs. nurse	2.97	1.55	5.67	0.001*	2.20	1.33	3.63	0.002*	2.82	1.52	5.24	0.001*	3.07	1.48	6.37	0.003*
Doctor vs. nurse	2.78	1.70	4.55	<0.001*	2.12	1.48	3.04	<0.001*	3.45	2.18	5.46	<0.001*	3.44	1.94	6.10	<0.001*
Private vs. public hospital	2.92	1.88	4.54	<0.001*	2.30	1.68	3.16	<0.001*	2.64	1.74	3.99	<0.001*	1.07	0.64	1.80	0.787
**Work hours (hour/week):**
70–100 vs. <70	1.89	1.32	2.72	0.001*	1.74	1.29	2.33	<0.001*	1.64	1.17	2.29	0.004*	1.21	0.77	1.89	0.407
>100 vs. <70	3.83	1.86	7.90	<0.001*	3.17	1.65	6.09	0.001*	2.38	1.19	4.76	0.015*	0.75	0.25	2.28	0.611
Emergency service	1.11	0.75	1.66	0.598	1.15	0.84	1.57	0.379	1.28	0.89	1.85	0.189	0.78	0.49	1.24	0.291
Non-emergency service	0.82	0.54	1.24	0.351	1.22	0.88	1.70	0.232	1.24	0.85	1.81	0.271	1.18	0.72	1.94	0.505
Administrative	0.39	0.16	0.94	0.035*	0.73	0.39	1.35	0.315	0.41	0.18	0.92	0.032*	1.54	0.70	3.43	0.285

*EE, Emotional exhaustion; DP, depersonalization; Personal accomplishment; OR, Odds ratio; 95% CI, 95% Confidence interval.*

*Significant p-value at <0.05 (bold).

**Figure 1:**
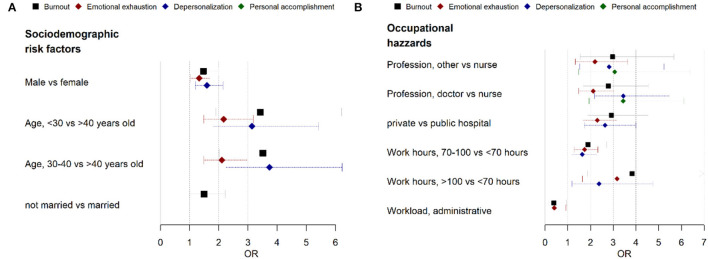
A logistic regression plot of odds ratio (*OR*) and 95% confidence interval (*CI*) of the burnout subscale and the associated factors. **(A)** Sociodemographic factors and **(B)** occupational hazards.

Exhaustion and cynicism can be observed in respondents who have younger age, male gender, medical profession, worked in private hospital, and long work hours. Respondents with an age of under 30 years and those aged from 30 to 40 years tend to experience a higher level of both exhaustion by 2-fold and cynicism by 3-fold compared with respondents more than the age of 40 years (*p*-value < 0.001). Being male health professionals in Malang may also contribute to the higher level of EE by 1.3-fold (*p*-value = 0.022) and DP by 1.6-fold (*p*-value = 0.001) compared with the female health professional. Moreover, an association was observed on longer work hours increasing exhaustion risk by 3-fold (*p*-value = 0.001) and cynicism risk by 2-fold (*p*-value = 0.015) on respondents who worked more than 100 h/week. For hospital sector, our observation showed health professionals who worked in private hospitals tend to have a high-level of EE and DP (*p*-value < 0.001). Furthermore, doctors as a medical profession have an increasing score in all burnout subscales concurrently, such as higher EE, higher DP, and lower PA (*p*-value < 0.001). Compared with a nurse, others health professions also showed similar results with higher EE, higher DP, and lower PA (*OR* = 2.20, *p*-value = 0.002; *OR* = 2.82, *p*-value = 0.001; and *OR* = 3.07, *p*-value = 0.003). Those results are presented in Table 3 and are visualized with a forest plot in Figure 1.

## Discussion

During the COVID-19 pandemic, ~22.0% of health professionals in Malang suffered from burnout. Those numbers were smaller compared with burnout global prevalence due to the COVID-19 pandemic presenting 51.4% of health professionals from 60 countries around the world using one subjective question about burnout (22). Moreover, a similar result from Italy and Egypt showed that the prevalence rate of health professional burnout was 24.7–37.0% and 28.2–31.8%, respectively, if EE or DP score was used to determine burnout (23, 24). Similarly, in several Asian countries, such as China and Malaysia, there is a prevalence of burnout of 12.0–37.0% and 22.0–38.4%, respectively (25, 26). In Indonesia, Sunjaya et al. observed the prevalence rate of emotional fatigue due to COVID-19 was 26.8% during the early period of the COVID-19 pandemic (27). However, the differences between the prevalence rates given above were caused by several factors, such as differences in time of the survey, type of the respondents, and how the country handled the pandemic (28).

Our study has shown that the sociodemographic and occupational hazards that were associated with burnout were age, work hours, profession, and hospital sector. This finding was supported by several previous studies. Spanish and Argentinian studies showed health professionals with an age ranging from 31 to 40 years old and age >40 years have lower MBI-HSS score compared with younger health professionals (*OR* = 0.56, *p*-value = 0.019 and *OR* = 0.43, *p*-value = 0.040) (29, 30). However, work hours as a burnout factor in the study by Giusti et al. showed that there were increasing MBI-HSS scores associated with longer work hours although the respondents worked shorter, with an average of 25.8 (±16.8) h weekly than our respondents (31). Moreover, the number of studies about hospital sectors associated with burnout during the COVID-19 pandemic is still low but the evidence showed that the burnout rate of health professional in a private hospital in Indonesia was higher than in a public hospital (32). While for the profession, the weight of our data is still skewed toward 2 respondent types, doctors and nurses. The most prominent association of low PA in our study was found in the doctors, and similar results were also shown by the study conducted by Sevinc et al. by comparing the PA of anesthesiologists and nurses in the ICU during the COVID-19 pandemic (33). Eventually, the definition of health professionals in our study has a broad definition. Our result may show evidence between burnout conditions and other health professions, such as pharmacist, dietitian, and lab assistants, but it is not specifically divided and must be interpreted cautiously.

Our results showed that the other factors, such as marital status, gender, and workloads associated with burnout, but they still have inconsistent results. Our results and the result of the study conducted by Patel et al. show an increase in burnout conditions for unmarried health professionals, whereas Hu et al. and Duarte et al. studies show that married health professionals are less susceptible to burnout (34–36). However, when we compared the data about gender, more studies showed female health professionals were more susceptible to burnout, but our result showed the opposite (29, 30, 36). In Indonesia, our data suggest that male gender was more susceptible to burnout, and it was similar with the result from the study conducted on Jordanian Health professionals (37). We believe the diversity of work culture among countries may have affected this result. The Indonesian government regulates healthcare professionals to work 40 h/week, but more resources were needed when COVID 19 emerged. Patients with COVID-19 that came to hospital in Malang exceed the capacity of the COVID-19 emergency room and isolation wards until they were willing to spend the night in front of the hospitals. The number of patients had forced hospital management to deploy more manpower to the COVID-19 services. Unfortunately some health workers still had to continue their daily routine services after working in the COVID-19 ward. Meanwhile, Indonesian health professionals may also have long work hours because of multiple workloads. For example, a doctor may work on the emergency service, provide non-emergency care, and participate in the hospital management at the same time. Our result presented the data about how the COVID-19 pandemic may expose all types of healthcare services, but front-liners who work intensely with direct interactions with patients, in an emergency or non-emergency service, clearly show burnout clinically but not statistically. New evidence in our result shows that health professionals with the administrative task may decrease the odd of burnout with low EE and DP score in an uncertain way, and further observation must be made.

It is worth debating how each of the MBI-HSS subscales involves the burnout in our respondent during the COVID-19 pandemic. EE is the most important subscale to determine the burnout condition, and aging has a negative correlation with the EE subscale. The explanation behind this phenomenon may be affected by how younger health professionals thought toward the fairness in a workplace (38). Younger health professionals may be more susceptible to EE than the older adults because they are more influenced by the outcome they receive, such as benefit and compensation. Furthermore, another factor associated with burnout that we found was long work hours. Earlier studies have shown how long work hours can make health professionals have limited time to rest (39). Meanwhile, a high DP subscale from the health professionals often associated with physiological distress (40). Although the causes of distress in our respondents cannot be observed clearly, a previous study conducted by Babore et al. has shown that the COVID-19 pandemic has increased distress for health professionals (41). This condition may affect the decline in professionalism and empathy of health professionals, especially doctors (40). The phenomenon above is also supported by the low level of professional accomplishment in this group. Earlier studies have shown that a high PA score may be affected by a person's knowledge and the skills contributing to their work (42). Hereafter, the reason behind the associations above still cannot be explained clearly, and further exploration must be made.

Indonesian health professionals have been struggling to fight against the COVID-19 pandemic for almost 2 years when this study was written. The second wave of pandemic peaked on July 2021 with the highest COVID-19 incident rate of 50,000 people in a day and, in August 2021, Indonesia mourned over 1,777 deaths in a day (28). Those numbers gave burden to the Malang's health professionals psychologically and were recorded in our study. Our results showed a considerable portion of health professionals suffered from EE due to occupational hazards. However, the explanation behind this phenomenon is still obscure, but no one will be prepared with the fear of a disease that can spread and kill people in time, and it will affect the people working in the sectors (41). The results of our study confirm that burnout does occur among health professionals. If the numbers in our study continue to grow due to predictable factors, then things may get worse. Directly, burnout will increase the error rate made by a health professional (21). Coping stress mechanism by individuals is mandatory to fight the physiological burden among health professionals, but it usually depends on their unprotected free time. To treat the fear and horror, it will take more than just the readiness of the individuals. The larger groups, such as hospitals, an organization that provide health services, and locals or national government will also need to be prepared. The lack of supervision on current work regulations should be fixed to ensure that health professionals may use their rights in COVID-19 services. The providers must be able to protect the vulnerable individuals and may also give an access to healthcare workers who are exhausted from their work in a pandemic situation to have psychological support and intervention without discrimination and stigma (43).

There are several limitations that we found in our study. First, our study has not been able to show the prevalence of burnout without excluding the confounders because burnout may arise from many underlying factors, such as depression, anxiety, and an excessive level of fear of COVID-19 (11). Second, the prevalence of burnout in our study may differ from the prevalence in other studies. We believe this is caused by the diversity of hospital work culture and the various definitions of burnout that do not have global consensus definition yet (44). Burnout is associated with psychological conditions, so a direct diagnosis from a psychiatrist or clinical psychologist will provide enhancement in this field of research (45). Third, long work hour is an important factor associated with burnout in our study, yet the definition of long work hours associated with burnout remains unclear. Our previous study in Malang and other similar studies used 70 h/week as a cut-off point, but several studies showed that working >55 h/week was associated with medical conditions (17, 46–49). Those gaps made our result may differ in studies with a different work hour classification, and a standardized work hour classification was needed in future studies to assess the true effect of the association between work hours and burnout. Finally, we are aware that the factors that can be associated with burnout are still broad, such as income levels, interaction time with COVID-19 patients, compensation provided by the government, and other factors (50).

## Conclusion

Our study showed that approximately one-fifth of health professionals in Malang suffered by the COVID-19 pandemic burnout. Many factors may increase the burnout condition, but the factors of age and long work hours show a strong association if compared with other factors. In our study, other factors, such as male gender, younger age, not in marital commitment, medical profession, and working in a private hospital also associated with burnout condition. These factors need to be examined and discussed further to prevent burnout among health professionals and increase the success rate of COVID-19 management.

## Data Availability Statement

The raw data supporting the conclusions of this article will be made available by the authors, without undue reservation.

## Ethics Statement

The studies involving human participants were reviewed and approved by Ethics Committee of Saiful Anwar General Hospital. The patients/participants provided their written informed consent to participate in this study.

## Author Contributions

Idea and concept: BD and FP. Design, control, supervision, and critical review: BD, FP, and JF. Data collection and processing: BD, MI, and JK. Analysis and interpretation: BD, MI, JK, and JF. Literature review: BD, FP, and JK. Writing the article: BD, FP, and MI. All authors contributed to the article and approved the submitted version.

## Conflict of Interest

The authors declare that the research was conducted in the absence of any commercial or financial relationships that could be construed as a potential conflict of interest.

## Publisher's Note

All claims expressed in this article are solely those of the authors and do not necessarily represent those of their affiliated organizations, or those of the publisher, the editors and the reviewers. Any product that may be evaluated in this article, or claim that may be made by its manufacturer, is not guaranteed or endorsed by the publisher.
